# Educating nursing students to meet the mental health needs of vulnerable populations: a narrative review

**DOI:** 10.3389/fpubh.2026.1801434

**Published:** 2026-05-08

**Authors:** Therese L. Mathews, Andrew Lim, Stephanie Burge, Jayme Weber, Suzanne Nuss, Lynne Buchanan

**Affiliations:** College of Nursing, University of Nebraska Medical Center, Omaha, NE, United States

**Keywords:** mental health needs, nursing education curriculum, social determinants of health, undergraduate and graduate, vulnerable populations

## Abstract

Vulnerable populations are individuals at increased risk for health disparities due to interacting structural, social, and individual factors. Mental health needs are among the most consequential yet inconsistently addressed dimensions of care within vulnerable populations. While the nursing profession strives to provide equitable health care for all individuals, many nurses feel unprepared to respond to their needs. This narrative review examined the nursing education literature to identify methods, content, outcomes, and gaps in curriculum for vulnerable populations’ mental health needs. Among the 23 articles identified, most articles focused on children/adolescents, LGBTQ+, military/veterans, and individuals exposed to trauma. Active teaching strategies were the most common curricular approaches. Most studies reported improvement in knowledge, confidence, or self-efficacy. Trauma-informed care was less represented and evaluated than other areas.

## Introduction

Vulnerable populations in health care encompass a wide range of communities, with definitions varying depending on the scope and nature of the discussion. To date, no single, universally accepted definition of vulnerable populations exists. However, several concept analyses (1, 2), and a concept synthesis (3) have been conducted on the terms *vulnerable* and *vulnerability* in health care, specifically in nursing science. Across these definitions, similar characteristics emerge, in that vulnerable populations describe a group of people who are at increased risk for health disparities (4) based on intrinsic and extrinsic factors that work synergistically to affect health outcomes, whether positively or negatively (2, 3). The literature has provided numerous examples of vulnerable populations, including but not limited to the very young, the very old, individuals with disabilities, racial/ethnic minorities, sexual and gender diverse individuals, incarcerated individuals, and rural individuals (4). For the purposes of this review, vulnerable populations are operationalized consistently with this definition, identifying groups at increased risk for health disparities due to overlapping social, structural, and individual factors.

Vulnerable populations are disproportionately encumbered by social determinants of health (SDOH), with mental health needs among the most overlooked in health care delivery, despite being highly relevant to individual well-being. In one study, 45% of individuals identified as “vulnerable” in a primary care setting had a mental health disorder (5). However, it is important to note that mental health needs do not necessarily indicate the presence of a diagnosable mental disorder, as mental health encompasses well-being and functioning in addition to mental illness (6). In this review, mental health needs refer to psychosocial distress, trauma-related concerns, substance use risk, suicide risk, therapeutic communication needs, and related assessment and supportive care competencies encountered in general nursing practice.

Although nurses receive education in mental health, this preparation may not occur within the specific context of caring for vulnerable populations. Nurses are well-positioned to support the needs of vulnerable groups due to their preparation in holistic assessment and lifespan-based care; yet many nurses report feeling unprepared to address mental health needs, particularly among vulnerable populations (7).

The American Association of Colleges of Nursing (AACN) Essentials (8) provide a competency-based framework for scaffolding nursing curricula and evaluating how educational content prepares students for practice. In this review, eight AACN concepts of clinical judgment; communication; compassionate care; diversity, equity and inclusion (DEI); ethics; evidence-based practice (EBP), health policy, and social determinants of health (SDOH) were used as an interpretive lens for examining the educational approaches represented in literature. This approach is also consistent with World Health Organization (WHO) guidance on integrating mental health care into pre-service medical and nursing curricula through competency-based educational design (9). To further guide the practice of caring for those with mental illness, a recent position paper (10) purported that concepts of DEI most closely linked with best practices in mental health care.

While nursing education allows for specialization in psychiatric care, mental health care is relevant across virtually all patient populations regardless of psychiatric diagnosis and is especially important when caring for vulnerable populations. The specific question this review seeks to answer is: What educational content and curricular approaches are used in general undergraduate and graduate nursing programs to prepare students to identify and address the mental health needs of vulnerable populations?

## Methods

A narrative review methodology was selected to examine the current body of literature and provide an interpretive synthesis of how nursing students are prepared to address the mental health needs of vulnerable populations. In contrast to a scoping review, which aims to map the breadth of literature without critical appraisal, and a systematic review, which requires strict *a priori* protocols, a narrative review offers methodological flexibility and is well-suited to research questions that require interpretive synthesis, critical evaluation of educational pedagogy, identification of gaps, and the integration of the authors’ collective expertise to propose future directions (11). This review was informed by Sukhera’s (11) framework for rigorous narrative review and Theile and Beall’s (12) overview of the process.

### Inclusion and exclusion criteria

Articles were eligible for inclusion if they: (a) addressed undergraduate and/or graduate nursing education; (b) examined any educational content including specific educational program, course material, curricular content, or curriculum development related to the mental health needs of vulnerable populations; and (c) focused on vulnerable populations, defined as groups at increased risk for health disparities, including but not limited to older adults, LGBTQ+ individuals, women, children, racial/ethnic minorities, immigrants/refugees, individuals with low socioeconomic status, veterans, and survivors of human trafficking. Both empirical research and review articles were eligible for inclusion, as were interprofessional education studies with nursing representation and articles addressing social determinants of health (SDOH) or global health contexts.

Exclusion criteria included articles unrelated to nursing education, dissertations, conference abstracts, commentaries, and publications older than 10 years. Studies focused specifically on psychiatric or mental health nursing specialty courses were excluded because the purpose of this review was to examine how mental-health related competencies are addressed in general nursing education rather than in courses or programs where psychiatric content is the primary curricular focus. Articles focused on populations defined primarily by diagnosed mental health disorders were also excluded for the same reason.

### Search strategies

A university librarian conducted the literature searches in consultation with the authors. Three sequential searches were conducted from September to December 2025 across three databases (i.e., Embase, CINAHL, PubMed) and Google Scholar. The first search was used to assess the breadth of the literature and refine eligibility criteria, while the second and third searches were conducted using the revised search terms and inclusion criteria. Keywords and subject headings included variations of *nursing education*, *mental health*, and *vulnerable populations*. Additional population-specific terms (i.e., older adults, LGBTQ+ individuals, women, children, etc.) were added in the second and third searches to capture populations that may not be indexed under broader vulnerable population terminology. The search strategies are provided in the Supplementary materials.

**Figure 1:**
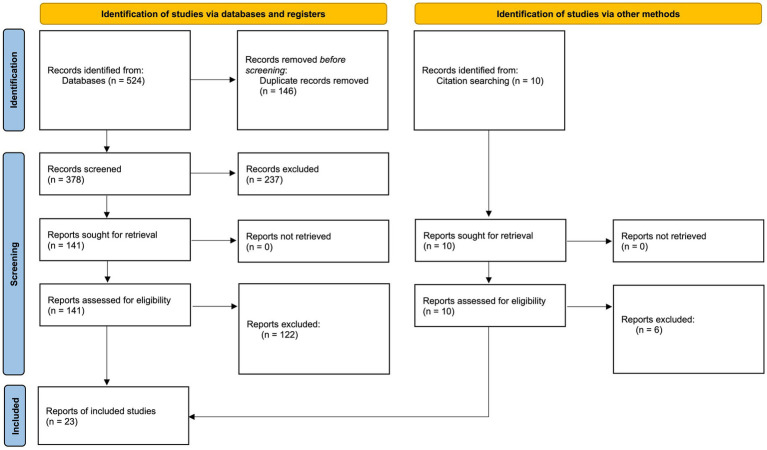
Flow diagram of the search process, adapted from the PRISMA 2020 statement (50).

Search results were exported to Zotero (version 8.0.5; Digital Scholar) for de-duplication. After duplicates were removed, 388 articles underwent title and abstract screening using the established inclusion and exclusion criteria. Screening and full-text review were conducted collaboratively by the six-author team, with each member independently reviewing assigned batches of articles before meeting to discuss and resolve discrepancies regarding final inclusion. Disagreements were resolved through team discussion and consensus. Title and abstract screening resulted in 151 articles for full-text reviews. After full-text review and final relevance assessments, 23 articles were retained for analysis. The search and screening process is illustrated using a flow diagram adapted from the PRISMA 2020 statement (Figure 1) (50).

### Data analysis

The included articles were analyzed using an approach consistent with Sukhera’s framework of critical evaluation, synthesis, and interpretation of findings (11). This process involved critically evaluating the methodology, data collection, and outcomes of each article. Relevant study characteristics were extracted and organized by vulnerable population, location, level of education, nursing concepts, pedagogical approach, and outcomes. Findings were then synthesized to identify key themes, patterns, and commonalities across studies. The studies were further interpreted by examining the potential relevance of outcomes comparing nursing concepts addressed and the mode of educational delivery. Finally, based upon the articles reviewed, gaps emerging from this review were identified. Research design and level of evidence were evaluated using the Johns Hopkins Nursing Evidence Appraisal Tool (13). Table 1 presents the included articles grouped by vulnerable population, location, research design, and evidence level.

**Table 1:** Articles categorized by vulnerable population, location, level of education, research design & level of evidence.

United States			
Population	Author/year	Research design	Evidence
Children/adolescents	Adamshick et al. (2024) (14)	Quasi-experimental	Level II
	Gill et al. (2019) (15)	Non-experimental	Level III
	Seigart et al. (2018) (16)	Quasi-experimental	Level II
	Treme et al. (2022) (17)	Non-experimental	Level III
	Hitchcock et al. (2019) (18)	Quasi-experimental	Level II
	Rohn et al. (2024) (19)	Quasi-experimental	Level II
LGBTQ+	Day et al. (2023) (22)	Non-experimental	Level III
	Stockmann and Diaz (2017) (24)	Quasi-experimental	Level II
	Vance et al. (2017) (25)	Quasi-experimental	Level II
	Vance et al. (2018) (26)	Quasi-experimental	Level II
	Maruca et al. (2018) (23)	Quasi-experimental	Level II
Veterans	McMillan et al. (2017) (27)	Non-experimental	Level III
	Rose et al. (2020) (28)	Non-experimental	Level III
	Magpantay-Monroe et al. (2017) (29)	Non-experimental	Level III
SDOH & Unspecified	Hartman and Phillips (2020) (32)	Non-experimental	Level III
	Mays et al. (2025) (33)	Non-experimental	Level III
	Singleton (2017) (34)	Non-experimental	Level III
Violence exposure	Burton et al. (2019) (30)	Non-experimental	Level III

International				
Population	Author/year	Country	Research design	Evidence
Children/adolescents	Semerci and Savas (2023) (21)	Turkey	Quasi-experimental	Level II
	Ozcevik Subasi et al. (2024) (20)	Turkey	RCT	Level I
Elderly/dementia	Shin & Lee (2019) (35)	Korea	Quasi-experimental	Level II
Perinatal depression	Nisar et al. (2022) (36)	China	RCT	Level I
Migrant/refugees	Muir-Cochrane et al. (2018) (37)	Australia	Mixed methods	Level III

## Results

Of the 23 included articles, 18 were conducted in the United States (US) and five were international. Four specific vulnerable population groups were represented in publications from the US: children and adolescents, LGBTQ+ individuals, veterans and military populations, and individuals exposed to trauma or violence. Three US studies did not specify a particular vulnerable population. International studies addressed children and adolescents, perinatal, older adults, and migrant or refugee populations.

### Vulnerable populations

#### Children and adolescents

Six US-based studies (14–19) and two international studies from Turkey (20, 21) addressed the mental health needs of children and adolescents Three (*n* = 3) articles incorporated adverse childhood experiences [ACEs; (14, 16, 18)], including a model evaluated using high-fidelity simulation (19). A scoping review found that case-based learning approaches were the most common and well-received methods of delivery of ACEs content (17). Three studies described virtual Screening, Brief Intervention and Referral to Treatment (SBIRT) which involved motivational interviewing for adolescent substance use prevention through combinations of lecture, simulation, and debriefing (14, 16, 18). The two international studies addressed therapeutic communication with hospitalized children (21) and knowledge of child abuse and neglect (20) through case presentations, guest lectures, video demonstrations, and interactive quizzes.

Across both US and international studies, the Essentials concepts of clinical judgment, communication, compassionate care, and EBP were consistently identified while SDOH and DEI were only apparent in one study (15). Multiple learning modalities which included passive and active learning strategies were used in five studies (14, 16, 18–21) with a notable improvement when debriefing with simulation was offered (14) while learning SBIRT skills.

Four studies reported positive outcomes in knowledge, confidence, readiness, communication skills and awareness (14, 15, 19, 21); however, changes in attitudes towards adolescents with substance use were limited (16, 18). Only one study (18) examined studied both undergraduate and graduate students, and both levels of students received the same SBIRT intervention with similar positive outcomes.

#### Lesbian, gay, bisexual, transgender, queer/questioning and other sexual and gender diverse individuals (LGBTQ+)

Five US-based articles focused on educating nursing students to meet the mental health needs of LGBTQ+ populations (22–26), with four specifically addressing transgender health (23–26). The educational interventions varied in both pedagogy and content. Educational interventions included an elective LGBTQ+ health course (22), transgender youth curriculum modules (25, 26), clinic observation (25), and simulation (23, 24). Across these studies, most of the AACN Essentials concepts (clinical judgment, communication, compassionate care, DEI, EBP, and SDOH) were consistently represented, while concepts of ethics and health policy were addressed less often. Content commonly addressed stress, suicide/self-injury, substance use, intimate partner violence, eating disorders (22), anxiety (22–24), gender dysphoria, and psychosocial assessment (25, 26).

Only one study used a validated instrument to measure intervention outcomes (23). Most studies reported gains in knowledge, awareness, or self-efficacy (22, 25, 26), and one multisite simulation study found improvement in affirmative practice behaviors but not attitudes (23). While observational experiences were beneficial, online modules were an effective standalone intervention. One study found that students felt unprepared to address the simulated individual’s mental health needs despite prior didactic exposure (24).

#### Veterans/military

Three US-based articles (27–29) focused on addressing the mental health needs of veterans and military populations in nursing education. Educational content included traumatic brain injury, post-traumatic stress disorder (PTSD), suicide prevention, combat experiences, and stress (27–29). These three studies addressed the concepts of clinical judgement, EBP, SDOH, and communication skills. Ethics and health policy were addressed in one study (28) and compassionate care was identified in two studies (28, 29). Teaching methods included clinical observation, structured debriefing, reflective writing, seminar discussion, simulation, guest speakers, and clinical experiences (27, 29). These studies identified several concerns, including student bias, confusion about best practices, and limited understanding of veteran-centric care (27, 29). A review of veteran-centric nursing competencies similarly found lack of standardization in content and competency-based evaluation tools (28).

#### Trauma/violence

One article addressed trauma-informed care (TIC) in undergraduate nursing curricula. Burton et al. (30) described strategies to increase TIC using the 2008 AACN Essentials framework (31), with all eight concepts used as guidelines for educating nursing students in respect to historical, structural, and interpersonal trauma, and community violence. Key assumptions of TIC were outlined, and illustrative examples of how TIC can be implemented were proposed. However, no outcomes were reported.

#### Unspecified vulnerable populations

Three articles addressed vulnerable populations more broadly through content focused on social determinants of health (SDOH), environmental justice, cultural competence, and health equity (32–34). All eight nursing concepts were identified in Hartman and Phillips (32). Four concepts (SDOH, DEI, EBP, and clinical judgment) were identified in Mays et al. (33), while two concepts (ethics and compassionate care) were identified in Singleton’s study (34). Multimodal learning strategies were incorporated across all three studies.

Students demonstrated improved ability to integrate cultural knowledge, communicate effectively, and engage in reflective practice, which contributed to stronger exam and licensure performance (32, 34). Curriculum that incorporated SDOH screening and culturally responsive practice also enhanced learners’ knowledge and attitudes, increased consideration of SDOH in clinical decision-making, and strengthened transcultural self-efficacy following the intervention (33, 34).

#### International research

Five international studies (20, 21, 35–37) addressed educational preparation of undergraduate nursing students to meet the mental health needs of vulnerable populations: two from Turkey [discussed in the children/adolescents section above; (19, 20)] and one each from Korea (35), China (36), and Australia (37). The three latter studies examined dementia care for older adults (35), psychosocial intervention education for perinatal depression (36), and mental health assessment in culturally diverse refugee and migrant populations (37). The concepts identified were clinical judgment (20, 36, 37), communication (20, 21, 35, 37), compassionate care (35, 37), DEI (37), and EBP (21, 36). Teaching methods varied widely and included lectures, case presentations, clinical experiences, e-training, role play, storytelling, and guided learning journeys (34–36). Reported outcomes included improved dementia knowledge and attitudes (35), and greater empathy and confidence in caring for patients with mental illness (37). There were no significant differences between electronic and face-to-face training methods for perinatal depression (36).

#### Emerging patterns

Across the included studies (*n* = 23), formal meta-analytic comparison was not feasible due to heterogeneity of populations, educational interventions, and outcome measures. However, several patterns emerged. Multimodal approaches were more common than single-method approaches and often combined didactic instruction with simulation, debriefing, case-based learning, clinical exposure, reflection, or other active strategies, particularly in child/adolescent, veteran, and broader SDOH-focused studies (14, 16, 18–21, 27, 29, 35–37).

Simulation and other active learning strategies seemed most useful for communication and practice-focused outcomes, including gains in confidence, readiness, knowledge, competence, and affirmative practice behaviors (14, 18, 23–26). However, attitudinal change appeared less consistently responsive to intervention, particularly in studies focused on adolescent substance use and LGBTQ+ affirming care (16, 18, 23), even when other outcomes improved. Positive outcomes were typically limited to self-reported knowledge, confidence, readiness, or satisfaction, whereas fewer studies assessed observable communication skills, counseling performance, or practice transfer (14, 19–26, 35–37).

When viewed through the AACN Essentials framework, the most represented concepts were clinical judgment, communication, EBP, and compassionate care, while DEI, SDOH, ethics, and health policy appeared less consistently and were usually embedded within broader curricular approaches rather than directly evaluated as outcomes. Trauma-informed care and population-specific counseling skills also remained underdeveloped within the review sample (25, 29, 30, 36).

## Discussion

This narrative review identified promising examples of educational interventions to prepare nursing students to meet the mental health needs of vulnerable populations. However, the findings should be interpreted cautiously because the evidence base was small, heterogeneous, and weighed toward nonexperimental and quasi-experimental designs. Within the included samples, most studies focused on a relatively narrow set of populations, particularly children and adolescents, LGBTQ+ individuals, and veterans. In contrast, little or no eligible literature was identified for several groups frequently described as vulnerable in health care literature, including survivors of human trafficking, racial and ethnic minorities, individuals with low socioeconomic status, incarcerated populations, and rural populations. This pattern may reflect true curricular gaps, limitations in indexing and terminology, or both.

The second major finding was that methodological rigor remains limited with only two randomized controlled trials identified within this review’s parameters. Many studies relied on small samples, single-site designs, or self-report measures. Positive outcomes were common, but they most often reflected self-perceived knowledge, confidence, or preparedness rather than directly observed communication skills, clinical judgment, or transfer to practice. Existing literature suggests that preparation related to mental health needs of vulnerable populations may receive less emphasis than other areas of nursing education (38), resulting in practicing nurses feeling unprepared to address mental health needs (7). The current literature suggests that educational interventions are feasible and often well received, but stronger designs and more robust outcome measures are needed to determine which approaches lead to lasting competence.

At the same time, this review identified encouraging findings. The combined use of didactic teaching and active learning was common across the literature and is consistent with broader literature supporting active learning for clinical reasoning and decision-making (39–42). Simulation, debriefing, observational experiences, and reflective activities appeared especially useful for translating sensitive content into practice-oriented learning. Nevertheless, some areas remained resistant to improvement. For example, changes in attitudes toward adolescents with substance use concerns were limited in some studies, and students continued to report uncertainty in counseling related to gender dysphoria, veteran-centric suicide risk, and perinatal depression despite the use of active learning strategies (16, 24, 29, 36). This may be especially relevant in the context of substance use, where stigma is shaped not only by knowledge deficit, but also by moral judgment and criminalization policy. Prior literature on educational interventions addressing stigma towards substance use disorders has reported mixed effects on attitudes, although immersion in clinical sites and critical reflection may offer some benefit (43, 44).

Viewed through the AACN Essentials, the included studies most often emphasized clinical judgement, communication, EBP, and compassionate care, suggesting that current educational efforts are oriented primarily toward interpersonal care and practice readiness. In contrast, SDOH, ethics, DEI, and health policy were less consistently represented or explicitly evaluated. Since DEI was identified as the most important concept in mental health care (10), it is paramount to include active learning strategies surrounding DEI in vulnerable populations. Examples may include acquiring more clinical training sites with diverse populations, immersion assignments, implicit bias training, and reflection of nursing core values particularly in respect to building interpersonal relationships and equitable care.

There was limited emphasis on trauma-informed care (TIC) in the literature identified in this review. Many vulnerable populations experience prior or ongoing trauma that is unrecognized, yet only one eligible article directly focused on TIC integration (30). This is notable because TIC is foundational to equitable, nonjudgmental, and psychologically safe nursing care. The limited evidence in this review suggests that TIC may be acknowledged conceptually but is not yet consistently operationalized, taught, and evaluated across nursing curricula. More intentional integration of TIC, including communication strategies and approaches that account for learners’ own trauma exposure, may strengthen both student preparation and patient-centered care.

It is important to acknowledge that relevant educational literature exists beyond the studies captured in this review. For example, recent nursing and interprofessional education scholarship has addressed vulnerable populations and related competencies through asynchronous online modules, interprofessional health disparities courses, poverty-focused experiential learning, and simulation-based teaching on social determinants of health (45–48). In addition, broader review-level work on integration of mental health competencies in undergraduate nursing education identified international literature not reflected in our sample (49). These examples suggest that the limited representation of some populations in the present review likely reflects, at least in part, the constraints of our search terminology and database retrieval rather than a definitive absence of relevant scholarship.

An interpretive boundary of this review is its focus on general nursing education rather than psychiatric or mental health nursing specialization (i.e., undergraduate mental health rotation, or PMHNP program). This distinction was intentional because the aim was to examine how undergraduate and graduate nursing programs prepare students for mental health needs that arise across practice settings and vulnerable populations. Therefore, the findings should be interpreted as reflecting the extent to which mental health-related competencies are incorporated into general nursing preparation rather than the full scope of mental health nursing education more broadly.

### Future directions

Future work should move beyond documenting exposure to vulnerable population content and instead identify which curricular approaches best support measurable competence in assessment, communication, clinical judgment, and referral. Studies should incorporate stronger outcome measures, such as observed performance in simulation, structured assessment of therapeutic communication, and evaluation of skill transfer into clinical settings.
