# Supplementary material for: Educating nursing students to meet the mental health needs of vulnerable populations: a narrative review
Source: Front Public Health. 2026 May 8;14:1801434. doi: 10.3389/fpubh.2026.1801434 (PMC13194560; doi:10.3389/fpubh.2026.1801434)
Supplement: Supplementary file 1 [file Data_Sheet_1.pdf]

Table 1. Summary of articles on vulnerable population mental health education in nursing students

Author, Year	Location	Level of nursing	Vulnerable population	Education delivery method	Education content	Key findings	Gaps identified in article
Adamshick & Payton, 2024	USA<br>PA	Undergraduate (n=79)	Adolescents	Simulation on SBIRT (Screening, Brief Intervention, and Referral to Treatment); asynchronous; 60 minutes.<br><br>Educational reinforcement (in-person debriefing) 2 days after posttest	SBI With Adolescents simulation developed by Kognito on substance use	Results showed mean scores for attitude, role security, knowledge, confidence, competence, readiness, and scenarios or cases improved significantly after the simulation ($p < .005$). Traditional undergraduate and postbaccalaureate students had similar posttest 1 and posttest 2 scores. The conclusion was that after SBIRT simulation, outcomes improved and were maintained after educational reinforcement.	More study to see if the change is sustained over time.
Burton et al., 2019	USA	Undergraduate	Violence/Trauma	Integration of Trauma Informed Care (TIC) into AACN Essentials (adding contents of social determinants of health [SDOH], trauma assessment, skills to evaluate research, proper documentation, legal requirements, sexual assault response team model, clinical experience, and simulation)	Forensic nursing<br>Historical Trauma<br>Structural Trauma<br>Violence/Abuse<br>Intimate Partner Violence (IPV)<br>Community violence<br>Adverse Childhood Experiences (ACE)<br>Trauma Informed Care	TIC necessary BSN content.<br><br>Commonly overlooked factor in health.<br><br>Ethical and holistic approaches must continue.	Gap in the number of specialty-prepared forensic nurses available
Day et al., 2023	USA<br>NY, PA	Both<br>Undergraduate (n=27)<br>Graduate (n=18)	LGBTQ+	Developed elective courses specific to LGBTQ+<br><br>SAIL (structured, active, in-person, learning) class, initially online due to COVID-19 surge then transitioned to in-person.<br><br>Pedagogy not described	Stress affecting gender and sexual minority groups<br><br>Mental health (suicide and self-injury; substance use; IPV; eating disorders; mood and anxiety)	2 elective courses (1 each at each institution) launched in 2022 and conducted online, which eventually transitioned to in-person.<br><br>Students' rationales for enrolling: recognizing self, lack of knowledge about LGBTQIA+ health issues, desiring to be better allies, personal sense of commitment to LGBTQ+ family and/or friends	Additional curriculum on LGBTQ+ concerns are needed.<br><br>More research on pedagogy.
Gill et al., 2019	USA<br>TN	Undergraduate	Children	Curriculum Integration model, scaffolding ACE concepts throughout five semesters of BSN curriculum	Develop awareness and prevent<br>Reduce toxic stress<br>Build resiliency/enable providers<br>Implement TIC<br>Inform Policy	Propose phases of curriculum integration and development.<br><br>Need systematic integrations	Integrate ACE in clinical courses.<br>Future research to evaluate outcomes.

Author, Year	Location	Level of nursing	Vulnerable population	Education delivery method	Education content	Key findings	Gaps identified in article
Hartman & Phillips, 2020	USA NC	Undergraduate (n=~288)	Various (refugees, incarcerated persons, persons experiencing homelessness, childbearing women at risk for postpartum depression, cognitive impairment)	Integrated mental health content across courses in four semesters: lectures (including guest lectures), simulation, standardized patients, case studies, community-based experience, inpatient clinical	Mental health screening, therapeutic communication, motivational interviewing, specific psychiatric disorders (bipolar disorder, depression, anxiety, eating disorder, schizophrenia, substance use disorder, cognitive impairment)	No significant difference in student scores of mental health standardized assessment between cohorts prior and after integrated curriculum. 3-year average NCLEX-RN first time pass rates remained constant.<br><br>Students were not aware of mental health concepts integration throughout the curriculum because they reported that faculty did not explicitly name each concept as mental health. Students also reported desiring more formal mental health class and time/opportunities to apply knowledge in clinical practice.	Need more visibility or explicit communication of integrated mental health objectives to students; no patient outcomes, just exam results; limited inpatient mental health exposure.
Hitchcock et al., 2019	USA AL	Both (n=108; 52% of which were nursing students; unclear how many were graduate)	Adolescents with substance use risk	Didactic (in-class lecture)<br><br>Simulation (SBI with Adolescents online simulation developed by Kognito; 3 practice conversations with adolescents)	SBIRT (Screening, Brief Intervention, and Referral to Treatment) model for adolescents; motivational interviewing skills; substance (alcohol and drug) use	Online simulation training improved students' confidence, perceived competence, and readiness to conduct SBIRT with adolescents at risk for substance use. No significant effect on attitudes and beliefs.	Limited learning outcomes. Need to assess actual knowledge and skills over time.
Magpantay-Monroe, 2017	USA HI	Undergraduate	Military/veteran	Didactic (lecture, EBP readings, informational materials); seminar (including guest speakers from military clinical partners); simulation (including military families); clinical placement with institutions specific to military/veterans and their families; reflection.	Stress and coping, anxiety disorders, obsessive compulsive, phobias, post-traumatic stress disorder (PTSD)	Students held some biases about the military/veteran population and thought that the population did not need unique care (standard care to civilians should be sufficient for all). Students lacked understanding about military culture (ethos, core values). Some students were not comfortable in a clinical agency with military/veteran patients.<br><br>Simulation experience provided insights and understanding to students, especially in military culture. Problem based learning project was helpful.	Include military and veteran health across curriculum.
Maruca et al., 2018	USA CT, FL	Undergraduate (n=47)	LGBTQ+	Didactic first (lecture) on LGBT content.<br><br>Simulation (manikin) specific to transgender patient. Debriefing after simulation.	LGBT content for lecture not described.<br><br>Simulation learning objectives: therapeutic communication, identifying s/s of anxiety, safely manage a client experiencing anxiety.	The median score of Gay Affirmative Practice (GAP) increased from 114 to 125, indicating improved affirmative practice. Furthermore, there was a statistically significant increase in the practice behavior subscale (but not in the beliefs/attitude subscale), indicating no effect on attitude.	Large number of those lost to follow-up.<br><br>GAP not specific to trans population.<br><br>Timing of study meant that participants only had minimal clinical experience in the past.

Author, Year	Location	Level of nursing	Vulnerable population	Education delivery method	Education content	Key findings	Gaps identified in article
Mays et al., 2025	USA<br>AL	Graduate<br>(n=521)<br><br>NP students	SDOH<br>Various (not defined)	Didactic and clinical over 3 semesters (SDOH screening and referral project).<br><br>Assignments included reflective essays, interviewing community members and preceptors, community assessment (using screening tools), writing literature review, writing a manuscript on intervention and outcomes	Foundational information on SDOH<br><br>Screening tool to assess SDOH needs in primary care settings (assesses food insecurity, housing, utilities, income, employment, transportation, education, substance abuse, childcare, safety, and abuse)	Statistically significant increases in self-assessed knowledge of SDOH; belief that addressing SDOH with patients is important; likelihood of considering SDOH when treating patients.<br><br>Students considered SDOH project as a valuable part of learning experience.	Organizational barrier: preceptor unwillingness, lack of clinical site<br><br>Structural barrier: lack of access to community resources<br><br>Personal barrier: students' biases, beliefs, and awareness of SDOH
McMillan et al., 2017	USA<br>AL	Undergraduate	Military/veteran	Project SERVE (Students' Education Related to the Veteran Experience) in community health population courses.<br><br>Didactic , forum, observational clinicals in different areas, panel discussions, guided de-briefs, final presentation.	Learning modules on TBI, PTSD, suicide prevention and combat stress; observational clinical experiences, debriefing, journals, reflection papers	Reflections written by students assisted with development of competencies for military care using the QSEN core competencies	This model can be used in nursing curricula along with further promoting immersion in clinical and community experiences
Muir-Cochrane et al., 2018	Australia	Both<br>Quantitative (n=43; all nursing);<br>Qualitative (n=11 nursing; n=10 others)	Migrant, refugees, aboriginal	"Guided learning journeys": four case studies that are self-paced in virtual environments. Components included audio, video, and images. Materials are open access.	Mental health assessment and treatment of specific vulnerable populations.	Quantitative: Empathy - increase in cognitive and affective empathy (r=0.62); Confidence - (r=0.67) (large effect); Cultural Competence - (r=0.47) (med-large effect size); all statistically significant.<br><br>Qualitative: positive comments, videos useful, increase use of critical thinking skills, "enlightening"	Further investigations needed in preparing nurses for cultural competence
Nisar et al., 2022	China	Undergraduate<br>(n=96)	Perinatal population in mid to low-income areas of China	e-learning training (videos, role plays, reflections, problem-solving strategies) vs. traditional face to face (control)	Psychosocial interventions for perinatal depression (counseling, collaboration with the mothers' families, guided discovery (of mental health attitudes), setting health-related tasks)	No significant differences between learning methods in competency, efficacy, satisfaction, attitudes and counseling skills compared to pre- to post testing.	Gap in mental health providers to deliver evidence-based instruction.

Author, Year	Location	Level of nursing	Vulnerable population	Education delivery method	Education content	Key findings	Gaps identified in article
Ozcevik Subasi et al., 2024	Turkey	Undergraduate (n=75)	Children & adolescents	Child Abuse and Neglect Awareness Program (CANAP) - developed and validated by the authors (didactic; case discussions; question-answer in online environment)	Four 40-min modules:<br>- Child neglect<br>- Child abuse<br>- Effects of abuse & neglect on children<br>- Child abuse & neglect prevention & responsibilities	CANAP effectively increases nursing students' CAN knowledge and awareness levels. But only physical symptoms knowledge was retained after one-month retest.<br><br>Experimental group's knowledge levels regarding the indicators of neglect increased immediately after the intervention (in the post-test) but returned to the same level 1 month later (in the re-test)	Targeted number of participants not achieved<br><br>Some students had taken a pediatrics course which may have influenced CAN knowledge<br><br>Lack of generalizability since conducted in one university<br><br>Frequency of post-tests (would have preferred re-test at 3 months)
Rohn et al., 2024	USA<br>MI	Undergraduate (n=129 surveys over 4 measurement points)	Children & adolescents	Didactic and simulation over 2 semesters.<br><br>Simulation adapted from a pre-existing commercially developed case.	Didactic: brain development; negative health outcomes linked to ACEs; trauma-informed care; resilience; policy<br><br>Simulation: caring for children with gunshot wounds (therapeutic communication; basic nursing tasks); caring for the same patient now in her 30s (mental health concerns)	Statistically significant improvement in ACEs knowledge (awareness & prevention, resilience, information policy)	Small sample size; no further examination of trauma-informed care understanding; short duration
Rose et al., 2020	USA<br>OR	Undergraduate	Military/veteran	Didactic (case studies, guest lectures)<br>Clinical	Various nursing care content relevant for veteran care identified through literature review. Mental health specific content included the incidence specific to veterans such as depressive disorders, bipolar disorders, anxiety disorders, schizophrenia, suicide, homelessness, and other psychotic disorders.	Veteran care content was integrated into 13 required undergrad courses; suicide prevention expert invited as guest speaker, simulations, and debriefing.	Evaluating the impact of veteran-care curricular content integration on student learning, and the faculty and student educational experience.

Author, Year	Location	Level of nursing	Vulnerable population	Education delivery method	Education content	Key findings	Gaps identified in article
Seigart et al., 2018	USA<br>ID	Undergraduate	Adolescent	Didactic (lecture)<br>Simulations (with standardized patients)<br>Clinical field experience (high school and university freshmen)	Using motivational interviewing in Adolescent SBIRT to screen, refer, and treat adolescents at risk.	Training is valued by students, faculty, and clinical partners. Faculty reported high degree of course content and skill retention in students.<br><br>Students have high level of satisfaction with the program.<br><br>No change in attitude towards substance users.	Increased workload for faculty to arrange interview experiences. High cost of commercial simulations used.
Semerci & Savaş, 2023	Turkey	Undergraduate (n=28)	Children (hospitalized)	Lecture, structured play activities, case presentations, video demonstrations, interactive quizzes; use of toys	Planned therapeutic communication activities to alleviate anxiety and establish trust; understand growth and development	Statistically significant decrease ($p < .001$) in non-therapeutic communication skills, and an increase ($p < .001$) in therapeutic communication skills.	Only a few studies about therapeutic communication (TC) skills and very few have been found to be effective. More research on methods of teaching TC is needed.
Shin & Lee, 2019	Korea	Undergraduate (n=122)	Elderly with dementia	Lectures, case presentations, clinical experience in senior recreational settings, foot massages, and art programs	Dementia theory education program on knowledge and attitudes of elderly care	Knowledge scores increased but not statistically significant. Attitude scores increase ($p = .027$), and elderly care attitude improved ($p = .003$)	Implementing prevention and management of dementia is insufficient. Manpower in nursing to serve dementia patients is needed.
Singleton, 2017	USA<br>NY	Graduate<br>(n=54)<br>DNP-FNP	"Vulnerable groups"	Online assignments and learning assessments; community resource repository; discussion board; power point presentations, articles	Enhanced cultural competence curriculum-specifically transcultural self-efficacy (TSE) (e.g., theory, systems & leadership, policy, ethical and legal, EBP, technology	Pre-/post test showed statistically significant increase ($p < .001$) for TSE. Experienced students with high TSE demonstrated large to very large gains in TSE after the curriculum. Between-group analyses reflected gains for each cohort showed a large gain for three cohorts: Cohorts 2, 3, and 5 had a gain of 1 SD above the mean, while Cohorts 1 and 4, had an after the curriculum. Between-group analyses reflected gains for each cohort showed a large gain for three cohorts: Cohorts 2, 3, and 5 had a gain of 1 SD above the mean, while Cohorts 1 and 4, had an extra large gain of 2 SD above the mean.	One program in NY limiting generalizability of study. Very diverse sample may not be representative of population in U.S. Self-report data. More research needed using objective assessment Measures to understand students' competence and confidence in transcultural nursing

Author, Year	Location	Level of nursing	Vulnerable population	Education delivery method	Education content	Key findings	Gaps identified in article
Stockman and Diaz, 2017	USA<br>FL	Undergraduate (n=20)	Transgender	High fidelity human simulation (HFHS) manikin<br><br>Prior: didactic course (on therapeutic communication, nurse-client relationship, anxiety)	Anxiety (identify symptoms, manage, use therapeutic communication)	Although there was a didactic course prior to the simulation experience, students still felt unprepared to address the simulated individual's mental health needs.<br><br>Students were able to identify and prioritize interventions for the simulated individual's anxiety, but having a partner during simulation helped as students themselves reported feeling anxious with the simulation.<br><br>Although students were able to identify their own vernal and nonverbal therapeutic communication skills, the manikin's lack of expression made the experience more challenging.	No explicit limitations were noted. However, it could be surmised that using a manikin had limitations in helping students identify and react to nonverbal cues.
Treme & Quick, 2022	USA<br>Scoping Review	Both	Children	Various (case based learning, preclinical assignments, lecture, role play activities, curricular content mapping, self-reflection)	Adverse childhood experiences (ACEs) and trauma-informed care (TIC)	A total of 22 articles were examined and included 15 research studies, two literature reviews, one quality improvement project, and four discussion articles.<br><br>Knowledge derived from this review includes safety of how to best incorporate content that is concentrated on safety issues for patients when these events are brought forth. Nursing students need to be educated on management and resources if this content is to be fully integrated into baccalaureate nursing curricula. This article also discussed research that needs to be done in this area to identify best practices for safe integration of content.	Lack of content on the safe and best way to integrate ACEs into the curriculum and the need for more research in this area.
Vance et al., 2017	USA<br>CA	Graduate<br>(n=4)<br><br>Also included students from other health professions (medical students, pediatric interns, psychiatry interns; n=16)	Transgender youth	Transgender Youth Curriculum consisting of online modules and observational experience.<br><br>The online modules consisted of six 15-min web modules covering a variety of topics related to transgender care. 2 hours were provided to complete.<br><br>Observational experience was a 5-hour clinic time.	Gender dysphoria<br><br>Other psychosocial history using HEADSS assessment	Knowledge and awareness of transgender youth medical and psychosocial considerations saw statistically significant improvement.<br><br>Learners were satisfied with overall and individual components of the curriculum.<br><br>Learners expected that curriculum materials would be useful in the future and intend to use information in the future.	Social desirability and recall bias were potential limitations. Self-assessment limited the objectivity of measurement. Future studies should develop more objective measures and analyze each individual curriculum component.

Author, Year	Location	Level of nursing	Vulnerable population	Education delivery method	Education content	Key findings	Gaps identified in article
Vance et al., 2018	USA<br>CA	Graduate<br>(n=6)<br><br>Also included students from other health professions (pediatric interns, psychiatry interns, medical students; n=30)	Transgender pediatric	Didactic. Consisted of six 15-minute online modules with a pre-module assessment.	Gender dysphoria<br><br>Other psychosocial history using HEADSS assessment	Learner's median overall objective knowledge score was statistically significantly increased. Statistical significance was also observed in the increased overall self-perceived knowledge score and self-efficacy scores.<br><br>Each individual self-efficacy item had a statistically significant increase, but the largest increase was in counseling transgender youth.<br><br>The online modules were seen as an effective standalone intervention.	Pre- and post-intervention testing is susceptible to response-shift bias.<br><br>Did not account for different learner types.
