# Supplementary material for: Educating nursing students to meet the mental health needs of vulnerable populations: a narrative review
Source: Front Public Health. 2026 May 8;14:1801434. doi: 10.3389/fpubh.2026.1801434 (PMC13194560; doi:10.3389/fpubh.2026.1801434)
Supplement: Supplementary file 2 [file Table_1.pdf]

Supplementary Materials

Search 1 MESH Terms

Embase:

('nursing education'/exp OR 'graduate nursing education':ti,ab OR 'master of science in nursing':ti,ab OR 'doctor or nursing practice':ti,ab) AND (train*:ti OR teach*:ti OR coach*:ti OR tutor*:ti OR curricu*:ti OR syllabus:ti OR module*:ti OR class*:ti) AND ('social determinants of health':ti OR 'lgbtqia people':ti OR 'socioeconomic status':ti OR homeless*:ti OR unhoused:ti OR rural:ti OR elder*:ti OR child*:ti OR infant*:ti OR adolescent*:ti OR teen*:ti OR 'pregnant woman':ti OR 'pregnant people':ti OR immigrant*:ti OR refugee*:ti OR 'mental health':ti) AND [2015-2025]/py AND [humans]/lim AND [english]/lim NOT 'conference abstract'/it

Google Scholar:

intitle:"graduate nursing education" OR intitle:"master of science in nursing" OR intitle:"doctor of nursing practice" AND train OR tutor OR curricula|curriculum OR class|classes AND "social determinants of health" OR lgbtqia+ OR "socioeconomic status" OR rural OR elder|elderly OR "mental health" OR child|children OR adolescent|adolescents OR infant|infants OR "pregnant woman|woman" OR "pregnant people" OR homeless|homelessness OR unhoused OR race OR minority|minorities OR immigrant|immigrants OR refugee|refugees

Search 2 MESH terms:

Embase (revised)

('nursing education'/exp OR 'graduate nursing education':ti,ab OR 'master of science in nursing':ti,ab OR 'doctor or nursing practice':ti,ab OR 'bachelor* of science in nursing':ti,ab OR 'undergrad* nursing degree*':ti,ab) AND (train*:ti OR teach*:ti OR coach*:ti OR tutor*:ti OR curricu*:ti OR syllabus:ti OR module*:ti OR class*:ti) AND ('social determinants of health':ti OR 'lgbtqia people':ti OR 'socioeconomic status':ti OR homeless*:ti OR unhoused:ti OR rural:ti OR elder*:ti OR child*:ti OR infant*:ti OR adolescent*:ti OR teen*:ti OR 'pregnant woman':ti OR 'pregnant people':ti OR immigrant*:ti OR refugee*:ti OR 'mental health':ti) AND [humans]/lim AND [english]/lim AND [2015-2025]/py NOT 'conference abstract'/it

Google Scholar (revised)

"graduate nursing education" OR "master of science in nursing" OR "doctor of nursing practice" OR “bachelor|bachelors of nursing” OR “undergraduate nursing degree” AND train OR tutor OR curricula|curriculum OR class|classes AND "social determinants of health" OR lgbtqia+ OR "socioeconomic status" OR rural OR elder|elderly OR "mental health" OR child|children OR adolescent|adolescents OR infant|infants OR "pregnant woman|woman" OR "pregnant people" OR homeless|homelessness OR unhoused OR race OR minority|minorities OR immigrant|immigrants OR refugee|refugees

CINAHL

(MH "Education, Nursing+" OR MH "Education, Nursing, Graduate+" OR XB(("graduate nursing education" OR "master of science in nursing" OR "doctor of nursing practice" OR “bachelor* of nursing” OR “undergraduate nursing degree”)) AND TI(train* OR teach* OR coach* OR tutor* OR curricu* OR syllabus OR module* OR class*) AND (MH "Social Determinants of Health" OR TI((“social determinants of health” OR “lgbtqia people” OR “socioeconomic status” OR homeless* OR unhoused OR rural OR elder* OR child* OR infant* OR adolescent* OR teen* OR “pregnant woman” OR “pregnant people” OR immigrant* OR refugee* OR “mental health”))

PubMed

("Education, Nursing"[Mesh] OR "Education, Nursing, Graduate"[Mesh] OR "Education, Nursing, Baccalaureate"[Mesh] OR "graduate nursing education"[tiab] OR "master of science in nursing"[tiab] OR "doctor of nursing practice"[tiab] OR "bachelor* of nursing"[tiab] OR "undergraduate nursing degree"[tiab]) AND (train*[ti] OR teach*[ti] OR coach*[ti] OR tutor*[ti] OR curricu*[ti] OR syllabus[ti] OR module*[ti] OR class*[ti]) AND ("Social Determinants of Health"[Mesh] OR "social determinants of health"[ti] OR "lgbtqia people"[ti] OR "socioeconomic status"[ti] OR homeless*[ti] OR unhoused[ti] OR rural[ti] OR elder*[ti] OR child*[ti] OR infant*[ti] OR adolescent*[ti] OR teen*[ti] OR "pregnant woman"[ti] OR "pregnant people"[ti] OR immigrant*[ti] OR refugee*[ti] OR "mental health"[ti]) AND ((y_10[Filter]) AND (humans[Filter]) AND (english[Filter]))

Search 3 MESH terms:

Embase

('nursing education'/exp OR 'graduate nursing education':ti,ab OR 'master of science in nursing':ti,ab OR 'doctor or nursing practice':ti,ab OR 'bachelor* of science in nursing':ti,ab OR 'undergrad* nursing degree*':ti,ab) AND (train*:ti OR teach*:ti OR coach*:ti OR tutor*:ti OR curricu*:ti OR syllabus:ti OR module*:ti OR class*:ti) AND ('social determinants of health'/exp OR 'social determinants of health':ti OR 'social driver* of health':ti OR 'health determinant*':ti OR 'lgbtqia people':ti OR lesbian:ti OR gay:ti OR bisexual*:ti OR transgender*:ti OR questioning:ti OR intersex*:ti OR asexual*:ti OR 'socioeconomic status':ti OR 'social class':ti OR 'social rank*':ti OR 'social station':ti OR income:ti OR job:ti OR jobs:ti OR occupation*:ti OR homeless*:ti OR unhoused:ti OR rural:ti OR countryside:ti OR pastoral:ti OR rustic:ti OR agricultur*:ti OR elder*:ti OR aged:ti OR mature:ti OR vulnerabil*:ti OR child*:ti OR infant*:ti OR adolescent*:ti OR teen*:ti OR woman:ti OR women:ti OR 'pregnant woman':ti OR 'pregnant people':ti OR immigrant*:ti OR refugee*:ti OR migrant*:ti OR 'mental health':ti OR 'mental health condition*':ti OR 'mental health disorder*' OR anxiety:ti OR depression:ti OR 'bipolar disorder*':ti OR 'post traumatic stress disorder*':ti OR ptsd:ti OR schizophrenia:ti OR

'dissociative disorder*':ti OR 'eating disorder*':ti OR 'disordered eating':ti OR anorex*:ti OR bulimi*:ti OR 'anorexia nervosa':ti OR 'bulimia nervosa':ti OR disable:ti OR disabilit*:ti OR 'hear* impair*':ti OR deaf:ti OR deafness:ti OR blind:ti OR blindness:ti OR 'vision impair*':ti OR 'impair* vision':ti OR 'low vision':ti OR intellect*:ti OR race:ti OR ethnic*:ti OR minorit*:ti OR 'african american*':ti OR hispanic:ti OR 'american indian':ti OR 'alaska native':ti OR 'native american*':ti OR 'asian american*':ti OR veteran*:ti OR 'chronic illness*':ti OR 'chronic disease*':ti OR 'long term illness*':ti OR 'long term disease*':ti OR 'persistant illness*':ti OR 'persistant disease*':ti OR prisoner*:ti OR inmate*:ti OR 'human traffick*':ti OR 'human smuggling':ti OR 'human sell*':ti) AND [humans]/lim AND [english]/lim AND [2015-2025]/py NOT 'conference abstract'/it

Google Scholar

"graduate nursing education" OR "master of science in nursing" OR "doctor of nursing practice" OR “bachelor|bachelors of nursing” OR “undergraduate nursing degree” AND train OR tutor OR curricula|curriculum OR class|classes AND "social determinants of health" OR lgbtqia+ OR lesbian|lesbians OR gay OR bisexual|bisexuality OR transgender OR questioning OR intersex OR asexual OR "socioeconomic status" OR “social status” OR “social rank” OR “social station” OR income OR job|jobs OR occupation|occupations OR rural OR countryside OR pastoral OR rustic OR agricultural OR elder|elderly OR aged OR mature OR vulnerable OR vulnerability OR "mental health condition|conditions disorder|disorders" OR anxiety OR depression OR “bipolar disorder|disorders” OR “post traumatic stress disorder|disorders” OR ptsd OR schizophrenia OR “dissociative disorder|disorders” OR “eating disorder|disorders” OR “disordered eating” OR anorexia OR bulimia OR “anorexia nervosa” OR “bulimia nervosa” OR disable|disability|disabilities OR “hearing impaired|impairment” OR deaf|deafness OR blind|blindness OR “vision|visual impaired|impairment OR “low vision” OR intellect OR race OR ethnicity OR child|children OR adolescent|adolescents OR infant|infants OR "pregnant woman|woman" OR "pregnant people" OR woman|women OR homeless|homelessness OR unhoused OR race OR minority|minorities OR immigrant|immigrants OR refugee|refugees OR minority|minorities OR “african american|americans” OR hispanic|hispanics OR “american indian” OR “alaska native” OR “native american” OR “asian american” OR veteran OR “chronic| long term|persistant illness||illnesses|disease|diseases” OR prisioner|prisoners OR inmate|inmates OR “human trafficking|smuggling|selling

CINAHL

(MH "Education, Nursing+" OR MH "Education, Nursing, Graduate+" OR XB(("graduate nursing education" OR "master of science in nursing" OR "doctor of nursing practice" OR “bachelor* of nursing” OR “undergraduate nursing degree”)) AND TI(train* OR teach* OR coach* OR tutor* OR curricu* OR syllabus OR module* OR class*) AND (MH "Social Determinants of Health" OR TI((“social determinants of health” OR “social driver* of health” OR “health determinant*” OR “lgbtqia people” OR lesbian OR gay OR bisexual* OR

transgender* OR questioning OR intersex* OR asexual* OR “socioeconomic status” OR “social class” OR “social rank*” OR “social station” OR income OR job OR jobs OR occupation* OR homeless* OR unhoused OR rural OR countryside OR pastoral OR rustic OR agricultur* OR elder* OR aged OR mature OR vulnerabil* OR child* OR infant* OR adolescent* OR teen* OR aged OR mature OR vulnerabil* OR “pregnant woman” OR “pregnant people” OR immigrant* OR refugee* OR migrant* OR “mental health” OR “mental health condition*” OR “mental health disorder*” OR anxiety OR depression OR “bipolar disorder*” OR “post traumatic stress disorder*” OR ptsd OR schizophrenia OR “dissociative disorder*” OR “eating disorder* OR “disordered eating” OR anorex* OR bulimi* OR “anorexia nervosa” OR “bulimia nervosa” OR disable OR disabilit* OR “hear* impair*” OR deaf OR deafness OR blind OR blindness OR “vision impair*” OR “impair* vision” OR “low vision” OR intellect* OR race OR ethnic* OR minorit* OR “african american*” OR hispanic OR “american indian” OR “alaska native” OR “native american*” OR “asian american*” OR veteran* OR “chronic illness*” OR “chronic disease*” OR “long term illness*” OR “long term disease*” OR “persistant illness*” OR “persistant disease*” OR prisoner* OR inmate* OR “human traffick*” OR “human smuggling” OR “human sell*”))

PubMed

("Education, Nursing"[Mesh] OR "Education, Nursing, Graduate"[Mesh] OR "Education, Nursing, Baccalaureate"[Mesh] OR "graduate nursing education"[tiab] OR "master of science in nursing"[tiab] OR "doctor of nursing practice"[tiab] OR "bachelor* of nursing"[tiab] OR "undergraduate nursing degree"[tiab]) AND (train*[ti] OR teach*[ti] OR coach*[ti] OR tutor*[ti] OR curricu*[ti] OR syllabus[ti] OR module*[ti] OR class*[ti]) AND ("Social Determinants of Health"[Mesh] OR "social determinants of health"[ti] “social driver* of health”[ti] OR “health determinant*”[ti] OR "lgbtqia people"[ti] OR lesbian[ti] OR gay[ti] OR bisexual*[ti] OR transgender*[ti] OR questioning[ti] OR intersex*[ti] OR asexual*[ti] OR "socioeconomic status"[ti] OR “social class”[ti] OR “social rank*”[ti] OR “social station”[ti] OR income[ti] OR job[ti] OR jobs[ti] OR occupation*[ti] OR homeless*[ti] OR unhoused[ti] OR rural[ti] OR countryside[ti] OR pastoral[ti] OR rustic[ti] OR agricultur*[ti] OR elder*[ti] OR aged[ti] OR mature[ti] OR vulnerabil*[ti] OR child*[ti] OR infant*[ti] OR adolescent*[ti] OR teen*[ti] woman[ti] OR women[ti] OR "pregnant woman"[ti] OR "pregnant people"[ti] OR immigrant*[ti] OR refugee*[ti] OR migrant*[ti] OR "mental health”[ti] OR “mental health condition*”[ti] OR “mental health disorder*”[ti] OR anxiety[ti] OR depression[ti] OR “bipolar disorder*”[ti] OR “post traumatic stress disorder*”[ti] OR ptsd[ti] OR schizophrenia[ti] OR “dissociative disorder*”[ti] OR “eating disorder*”[ti] OR “disordered eating”[ti] OR anorex*[ti] OR bulimi*[ti] OR “anorexia nervosa”[ti] OR “bulimia nervosa”[ti] OR disable[ti] OR disabilit*[ti] OR “hear* impair*”[ti] OR deaf[ti] OR deafness[ti] OR blind[ti] OR blindness[ti] OR “vision impair*”[ti] OR “impair* vision”[ti] OR “low vision”[ti] OR intellect*[ti] OR race[ti] OR ethnic*[ti] OR minorit* [ti] OR “african american[ti]” OR hispanic[ti] OR “american indian”[ti] OR “alaska native” [ti] OR “native american*” [ti] OR “asian american*” [ti] OR veteran* [ti]

OR “chronic illness*”[ti] OR “chronic disease*”[ti] OR “long term illness*”[ti] OR “long term disease*”[ti] OR “persistant illness*”[ti] OR “persistant disease*”[ti] OR prisoner*[ti] OR inmate*[ti] OR “human traffick*”[ti] OR “human smuggling”[ti] OR “human sell*”[ti]) AND ((y_10[Filter]) AND (humans[Filter]) AND (english[Filter]))
